# Dietary effects on gut microbiota of the mesquite lizard *Sceloporus grammicus* (Wiegmann, 1828) across different altitudes

**DOI:** 10.1186/s40168-020-0783-6

**Published:** 2020-01-24

**Authors:** Nina Montoya-Ciriaco, Selene Gómez-Acata, Ligia Catalina Muñoz-Arenas, Luc Dendooven, Arturo Estrada-Torres, Aníbal H. Díaz de la Vega-Pérez, Yendi E. Navarro-Noya

**Affiliations:** 1 Doctorado en Ciencias Biológicas, Centro Tlaxcala de Biología de la Conducta, Universidad Autónoma de Tlaxcala, Tlaxcala, México; 2 Laboratory of Soil Ecology, Cinvestav, Mexico City, Mexico; 3 Centro Tlaxcala de Biología de la Conducta, Universidad Autónoma de Tlaxcala, Tlaxcala, México; 4 Cátedras CONACyT, Universidad Autónoma de Tlaxcala, Tlaxcala, México

**Keywords:** Altitudinal gradient, Ectothermic vertebrate, Fecal microbiota, High-mountain ecosystem, Intestinal microbiota, Microbiome, Mycobiome

## Abstract

**Background:**

High-altitude ecosystems are extreme environments that generate specific physiological, morphological, and behavioral adaptations in ectotherms. The shifts in gut microbiota of the ectothermic hosts as an adaptation to environmental changes are still largely unknown. We investigated the food ingested and the bacterial, fungal, and protistan communities in feces of the lizard *Sceloporus grammicus* inhabiting an altitudinal range using metabarcoding approaches.

**Results:**

The bacterial phyla *Bacteroidetes* and *Firmicutes*, and the genera *Bacteroides* and *Parabacteroides* dominated the core fecal bacteriome, while *Zygomycota* and *Ascomycota*, and the species *Basidiobolus ranarum* and *Basidiobolus magnus* dominated the core fecal mycobiome. The diet of *S*. *grammicus* included 29 invertebrate families belonging to *Arachnida*, *Chilopoda*, and *Insecta*. The diversity and abundance of its diet decreased sharply at high altitudes, while the abundance of plant material and *Agaricomycetes* was significantly higher at the highest site. The composition of the fecal microbiota of *S*. *grammicus* was different at the three altitudes, but not between females and males. Dietary restriction in *S*. *grammicus* at 4150 m might explain the high fecal abundance of *Akkermansia* and *Oscillopira*, bacteria characteristic of long fasting periods, while low temperature favored *B*. *magnus*. A high proportion of bacterial functions were digestive in *S*. *grammicus* at 2600 and 3100, while metabolism of aminoacids, vitamins, and key intermediates of metabolic pathways were higher at 4150 m. Different assemblages of fungal species in the lizard reflect differences in the environments at different elevations. Pathogens were more prevalent at high elevations than at the low ones.

**Conclusions:**

Limiting food resources at high elevations might oblige *S*. *grammicus* to exploit other food resources and its intestinal microbiota have degradative and detoxifying capacities. *Sceloporus grammicus* might have acquired *B*. *ranarum* from the insects infected by the fungus, but its commensal relationship might be established by the quitinolytic capacities of *B*. *ranarum.* The mycobiome participate mainly in digestive and degradative functions while the bacteriome in digestive and metabolic functions.

## Background

The symbiotic relationship between vertebrate hosts and their intestinal microbiota is complex and has affected significantly the ecology and evolution of both [1]. Understanding the role of gut microbiota in the evolution of their vertebrate host is an outstanding question and the focus of much current research. The primary function of the gut is to obtain nutrients and gut microbiota play therein a crucial role, but they contribute also to the overall health of the vertebrate host. Experimental and comparative studies have found that gut microbial communities contribute to balancing energy, physiology, reproduction, immunity, organ development, behavior, and life history of the host [1–4]. Conversely, environmental factors, such as diet and population density, and host traits, such as infections and genetics, may affect the microbial gut communities [5–7].

Reptiles represent 17% of all vertebrate species and the order of *Squamata* contains almost 8000 species. Reptiles are ideal to determine the effect of temperature increases as a result of climate change and habitat degradation as they depend heavily on specific environmental conditions, i.e., they are ectotherms [8]. Ambient temperature determines characteristics of ectotherms, such as body size, reproduction, offspring, diet, metabolism, behavior, locomotion, and survival [9–11]. Ectotherms have a wide range of physiological adaptations that vary within members of the same species, but living in different conditions. Mountainous ecosystems provide a natural gradient of different environmental conditions as the temperature decreases with 0.6 °C per 100 m a.s.l., which allow to study the effect of these conditions on the gut microbial biota of ectotherms along the altitude gradient.

*Sceloporus grammicus* Wiegmann, 1828 (*Squamata* order), the mesquite spiny lizard, is an insectivore with a tendency to feed on *Coleoptera* and *Hymenoptera* [12]. The distribution of *S*. *grammicus* extends from southern Texas in the USA to southern Oaxaca in Mexico. It inhabits a diversity of environments ranging in altitude from 1500 to 4400 m a.s.l. and could be the most widespread and adaptable lizard in Mexico [12, 13].

In the National Park “*La Malinche*,” a volcano of the Trans-Mexican Volcanic Belt (TMVB), mesquite spiny lizards can be found up to 4200 m a.s.l. [14]. The body temperature of *S*. *grammicus* decreases with increasing elevation. These extreme environmental conditions impose a high cost of thermoregulation. A wide range of physiological and behavioral strategies allows this species to successfully inhabit high-mountain ecosystems [15]. Environmental temperature has been reported to affect gut microbiota in ectotherms. Kohl and Yahn [16] found that temperature affected significantly the microbial community structure in the gut of tadpoles. Bestion et al. [6] used a semi-natural experiment to study the effect of temperature on the gut microbiota of the *Zootoca vivipara* lizard and found a 34% diversity loss at high temperatures. Living at high altitudes also alters the availability of food that changes the lizards’ diet, which will also require physiological adaptations and will certainly affect the gut microbiota. The gut mass of animals is proportional to the altitude reflecting an adaptation of digestive and absorptive functions [17]. Zhang et al. [18] reported significant changes in the intestinal bacterial composition of the toad-headed lizard *Phrynocephalus vlangalii* from three different altitudes in the Tibetan Plateau*.*

However, we still lack an understanding of the role of exogenous factors on the gut microbiota in ectotherms in relation with their ecophysiology. These changes in the gut microbiota might aid the host in adapting to the high altitude environmental conditions. It can be assumed that changes in the gastrointestinal traits of evolving vertebrates selected for essential taxa altering the gut microbiome profiles [1]. The gut microbiota is composed mostly of bacteria, fungi, nematodes, and viruses. Fungi might play an important role in the food degradation considering the insectivorous diet of *S*. *grammicus*. However, the fungal component, the mycobiome, has received little attention compared to bacteria. In the gut of the highland lizards, fungal taxa might aid in the digestion of food while bacteria in nutrient adsorption.

In this study, we used metabarcoding approaches to study the bacterial, fungal, and protistan communities, and the ingested food in feces of *S*. *grammicus* living between 2600 and 4150 m a.s.l. in a high-mountain ecosystem. The functional profile prediction of the fecal bacterial communities was done by ancestral reconstruction of the bacterial taxonomic assemblage to obtain insights into the functionalities of the resident bacterial biota.

## Methods

### Pilot study

The study area was located in the territory of the National Park “*La Malinche*” (NPLM) (N 19°, 14′ W 98° 02′). Four adult individuals (two males and two females) were collected from the NPLM at 4150 m a.s.l. on 22th June 2015. Lizards were taken to the laboratory in Tlaxcala city, and maintained individually in sterile boxes until feces were obtained. Feces were collected in sterile conditions, added separately to 1.5 ml sterile polypropylene tubes, frozen immediately at − 20 °C, and extracted for DNA the next day. Individuals were dissected in sterile conditions and the gastrointestinal tract was collected and placed in a sterile tube.

All laboratory analyses were done under strict sterile conditions. The feces and gastrointestinal tracts were extracted for metagenomic DNA as follows. First, the gastrointestinal tract tissue was macerated with a sterile pistil in tubes containing 3 ml buffer (0.15 M NaCl, 0.1 M EDTA [pH 8.0]), weighted (varying between 126 and 580 mg), and divided equally over three tubes. Feces were washed twice with 1-ml decahydrated tetrasodium pyrophosphate 0.15 M and washed twice with phosphate buffer pH 8 0.15 M. Three different techniques were used to extract DNA from the samples. The first method consisted in a chemical and thermal shock of the cells [19]. Cells were enzymatically lysed in the second method [20], while a detergent solution and mechanic disruption for cell lysis was used in the third method [21]. This last method promotes the lysis of filamentous fungi and yeast. The DNA obtained from the three extraction methods was pooled so that one DNA sample was obtained and used for preparing the amplicon libraries. Blank controls were included in each extraction protocol. These negative controls were pooled and verified for contamination by gel electrophoresis and 16S rRNA PCR. Amplicon libraries of V3–V4 regions of 16S rRNA genes were obtained using the primers described by Klindworth et al. [22]. The 300-pb paired-end (PE) MiSeq runs (Illumina) were done by Macrogen Inc. (DNA Sequencing Service, Seoul, Korea).

The bacterial community composition of the gastrointestinal tract and feces were compared and as the species turnover was 46.9 ± 3.3%; no *S*. *grammicus* individuals had to be sacrificed to study their gut microbiota.

### Sites of study and fecal sampling

The lizards were collected at 2600, 3100, and 4150 m a.s.l. and considered the Low-2600, Medium-3100, and High-4150 zone. The Low-2600 zone is characterized by a temperate and semi-arid climate with mean air temperature of 14.5 ± 6.6 °C and mean relative humidity 58.2 ± 28.8% [15]. Cultivation of maize (*Zea mays* L.) was predominant in this area and lizards were collected from an abandoned building. Lizards in this zone weight on average 6.9 g and their mean snout to vent length (SVL) was 60.5 mm. The Medium-3100 zone is characterized by a semi-cold and sub-humid climate. The mean air temperature is 9.5 ± 5.4 °C and mean relative humidity is 73.8 ± 22.36%. The vegetation contained mainly pine (*Pinus montezumae* Lamb) and fir (*Abies religiosa* Kunth). Lizards in this zone weight on average 6.6 g and their SVL measure was 58 mm. The High-4150 zone was covered by alpine bunchgrasses (*Festuca* L., *Calamagrostis* Adans., and *Muhlenbergia* Shreb.) and is defined as a cold weather regimen [23]. This is a harsh environment for lizards and considered thermally restrictive, i.e., mean air temperature is 6.8 ± 6.6 °C and mean relative humidity is 77.8 ± 19.6%. Lizards of this zone weight 3.8 g and their SVL was 49.9 mm [15].

A total of 96 adults *S*. *grammicus* males and females were collected (Table 1). The lizards were collected manually, stored individually in sanitized boxes, and taken to the Research Station “*La Malinche*” (19° 14′ 38.6′′ N 97° 59′ 26.0′′ W; 3130 m a.s.l.) for fecal sampling. Lizards were marked on their leg scales with medical cautery unit to avoid recapture. At the research station, lizards were maintained at 20–25 °C individually in sterile boxes < 12 h with natural periods of day/night until feces were obtained from each individual. Feces were collected under strict sterile conditions. After collection, the feces were stored and transported on ice (< 4 °C) to the laboratory in Tlaxcala city. They were kept at − 20 °C for less than a week until extracted for DNA. After the feces were obtained, all lizards were released at their place of capture.

**Table 1:** Number of individuals of *Sceloporus grammicus* sampled per population

Population	Geographic location	Altitude	Date of sampling	Number of individuals
Low-2100	N 19° 12′ 32′′ W 97° 55′ 36′′	2,653	August 21–23 2015	23 Females
				22 Males
Medium-3100	N 19° 14′ 35′′ W 97° 59′ 25′′	3,124	October 18–20 2015	14 Females
				10 Males
High-4150	N 19° 14′ 03′′ W 98° 01′ 43′′	4,158	September 16–19 2015	18 Females
				9 Males
Total				55 Females
				41 Males

### Metabarcoding analysis of the 16S rRNA, 18S rRNA, and *coxI* genes and ITS region

The metagenomic DNA of the lizard feces was obtained by three lysis methods as mentioned before. The variable regions V1–V6 of 16S rRNA gene were amplified with primers 8-F (5′–CCA TCT CAT CCC TGC GTC TCT CCG–3′) and 949-R (5′–CCT ATC CCC TGT GTG CCT TGG CAG TCT CAG–3′) [19]. The 18S rRNA gene was amplified with the primers nu-SSU-0817 (5′–TTA GCA TGG AAT AAT RRA ATA GGA–3′) and nu-SSU-1196 (5′–TCT GGA CCT GGT GAG TTT CC–3′) [24]. The fungal internal transcribed spacer (ITS) ITS1-5.8S-ITS2 region was amplified with the primers ITS1F (5′–CTA CGG GIG GCW GCA G–3′) [25] and ITS4R (5′–GAC TAC HVG GGT ATC TAA TCC–3′) [26]. Additionally, 397 base pairs of the *coxI* were amplified with the primers mICOIintF (5′–GGW ACW GGW TGA ACW GTW TAY CCY CC–3′) [27] and jgHCO2198 (5′–TAI ACY TCI GGR TGI CCR AAR AAY CA–3′) [28]. All primers used contained the adapter for sequencing platform and 8 nt barcodes. Amplification reactions were done in quadruplicate, pooled, and purified using QIAquick PCR purification kit according to manufacturer’s instructions (QIAGEN Inc., Valencia, CA). Blank controls of PCR reagents and positive controls were included in each PCR batch. All PCR controls were pooled, purified, and included in a PCR assay to incorporate sequencing adaptors, but no amplicons were obtained. The quantification of the PCR products was done using a NanoDrop 3300 fluorospectrometer (Thermo Fisher Scientific, Waltham, MA, USA) with PicoGreen dsDNA assay (Invitrogen, Carlsbad, USA) and combined in equimolar quantities for sequencing by Macrogen Inc. Sequencing of 16S rRNA libraries was done with a Roche GS–FLX Plus 454 pyrosequencer (Roche, Mannheim, Germany), while fungal ITS and 18S rRNA and *coxI* genes with 300-pb PE MiSeq runs.

### Bioinformatics analysis

Sequence analysis was done in QIIME version 1.9.1 (available at www.qiime.org). Sequences were analyzed and filtered for quality parameters. Quality filtering was done based on the following criteria: no ambiguous base calls and quality values less than 23 Phred Q score. Paired-end sequences were assembled with fastq-join method within QIIME. Operational taxonomic units (OTUs) were determined at a similarity threshold of 97% (OTU-97%) with the open reference method of UCLUST [29]. Operational taxonomic units with less than two observations were eliminated. Representative sequences of each OTU-97% 16S rRNA sequences were aligned with database GreenGenes version 1210 available at http://greengenes.lbl.gov/Download/. The taxonomy assignment was done using the Ribosomal Data Project (htpp://rdp.cme.msu.edu/classifier.jsp) [30] with 80% confidence threshold. The SILVA database version 132 (available at https://www.arb-silva.de/downoload/archive/qiime/) was used for the analysis of 18S rRNA gene sequences. The non-redundant version of the UNITE+INSDC fungal ITS database [31] was used for analysis of the ITS region, while *coxI* taxonomic assignation was done with the BOLD ID Engine (available at http://v3.boldsystems.org/).

### Microbial diversity and statistical analysis

The equivalent Hill numbers were calculated with the matrices of OTU abundances. The alpha diversity profile of *q* = 0, 1 and 2 were obtained with the MetagenomeDiversity script in R [32]. All statistical analyses were done with R [33]. The distance matrix UniFrac of the microbial community composition using 16S rRNA and 18S rRNA genes was done using Fast UniFrac [34]. A Bray-Curtis distance matrix was determined for the fungal communities. The microbial community composition was explored by non-metric dimensional analysis (MDS) using the UniFrac and Bray-Curtis distance matrices, and to find differences in bacterial, fungal, and protist communities of the three populations of *S*. *grammicus*, permutational multivariate analysis of variance (perMANOVA) was done. perMANOVA tests were done with the vegan package [35]. Heat-maps were constructed with the pheatmap package [36]. Kruskal-Wallis and post-hoc Dunn’s test was used to determine the effect of altitude on the relative abundance of the different microbial groups of Bacteria, Fungi, and microscopic Eukaryote with the package FSA. Linear mixed effects models were done with the nlme package and probabilities were calculated with permutational analysis based on 1000 Monte Carlo samplings [33].

### Functional profile prediction of fecal bacterial communities of *Sceloporus grammicus*

The KEGG Orthologs functions of the metagenome were predicted using an ancestral state reconstruction algorithm with PICRUSt version 1.0.0 [37]. Briefly, OTUs of the 16S rRNA gene sequences were clustered at 95% similarity using the closed-reference strategy within QIIME and against the GreenGenes reference data base version 13.5 [38]. The OTU-table was normalized to correct the number of multiple 16S rRNA gene copies using the GreenGenes reference data base version 13.5. The database KEGG Orthology (KO) [39] was used to estimate functional genes in fecal bacterial biota of *S*. *grammicus*. The statistics and graphics were done in STAMP [40].

## Results

### Preliminary study: comparison of the gut and fecal bacterial biota of *Sceloporus grammicus*

A total of 250,916 high-quality reads were obtained with no *S*. *grammicus* mitochondrial gene. Eight biological samples of four *S*. *grammicus* individuals were analyzed and grouped into 1839 OTU-97%.

**Fig. 1:**
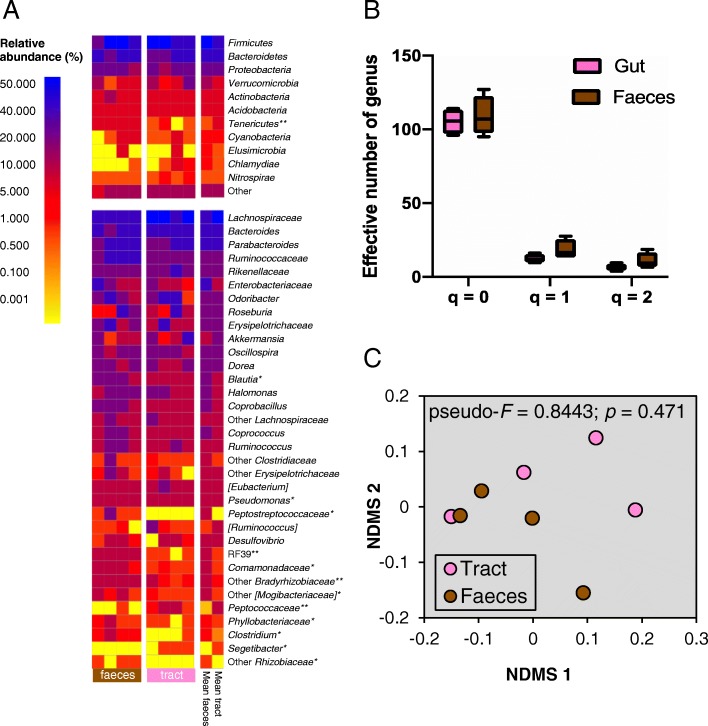
Bacteria in the gastrointestinal tract and feces of *Sceloporus grammicus* Wiegmann. Heat-map of the relative abundance of the most abundant taxonomic groups (**a**), Hill numbers at diversity *q* orders 0, 1, and 2 of the genera (**b**), and non-metric dimensional analysis (MDS) of the weighted UniFrac distances of the gastrointestinal tract and feces (**c**). Linear mixed effects models with 1000 Monte Carlo permutations and lizard identity as random factor (*p < 0.05, **p < 0.01) was used to test the significant differences on the relative abundance of the different bacterial groups and perMANOVA to find differences in bacterial communities in the gastrointestinal tract and feces

The bacterial gut biota of *S*. *grammicus* belonged to 30 different phyla, although 25 of them had relative abundance < 1%. *Firmicutes* and *Bacteroidetes* dominated the bacterial community of the gut and feces of *S*. *grammicus* with *Bacteroides* and *Parabacteroides* the most abundant genera (Fig. 1a). The relative abundance of *Peptococcaceae* and *Segetibacter* was significantly higher in the gastrointestinal tract than in feces, while the relative abundance of *Tenericutes*, *Peptostreptococcaceae*, *Clostridium*, *Phyllobacteriaceae*, *Bradyrhizobiaceae*, *Mollicutes* RF39, *Blautia*, *Pseudomonas*, and [*Mogibacteriaceae*] was significantly higher in the feces than in the gastrointestinal tract (*p* < 0.05). The effective numbers of bacterial genera at *q* = 0, 1, and 2 diversity orders were similar in the feces and the gastrointestinal tract (*q* = 0, *t* = 0.52, *p* = 0.638; *q* = 1, *t* = 0.62, *p* = 0.575; *q* = 2, *t* = 0.41, *p* = 0.711) (Fig. 1b). The bacterial community structure considering OTUs-97% was not different significantly between feces and the gastrointestinal tract as determined with the perMANOVA analysis of the weighted UniFrac distances (pseudo-*F* = 0.8443; *p* = 0.471) (Fig. 1c).

### Structure and composition of the fecal microbiota of *Sceloporus grammicus* along an altitudinal gradient

#### Bacterial communities

**Fig. 2:**
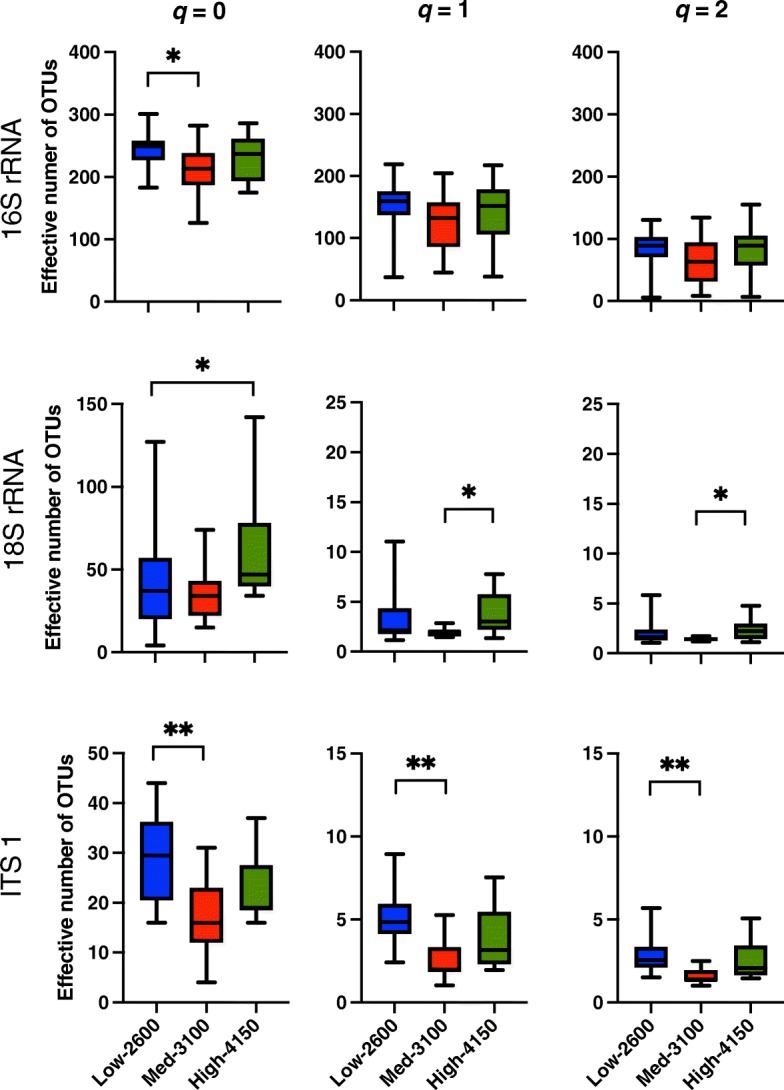
True diversity (Hill numbers) at diversity *q* orders 0, 1, and 2 of the grouped operational taxonomic units (OTUs) of the 16S rRNA gene, 18S rRNA, and ITS region of the fecal samples of *Sceloporus grammicus* Wiegmann found at 2600 m, 3100 m, and 4150 m. Significant differences between altitudes were tested by Kruskal-Wallis and post-hoc Dunn’s test (*p ≤ 0.05, **p ≤ 0.01, ***p ≤ 0.001)

A total of 85,480 high-quality sequences of 940 nt long of 16S rRNA gene were obtained and 2788 OTU-97%’s were clustered. The effective number of diversity order *q* = 0 (^0^D_α_) of Bacteria was 243 ± 26 in the Low-2600, 212 ± 38 in the Medium-3100, and 232 ± 35 in the High-4150 population (Fig. 2). The effective number of diversity order *q* = 1 (^1^D_α_) was 154 ± 34 in the Low-2600, 122 ± 47 in the Medium-3100, and 144 ± 49 in the High-4150 population, while the effective number of diversity order *q* = 2 (^2^D_α_) was 84 ± 27, 66 ± 39, and 84 ± 42, respectively.

**Fig. 3:**
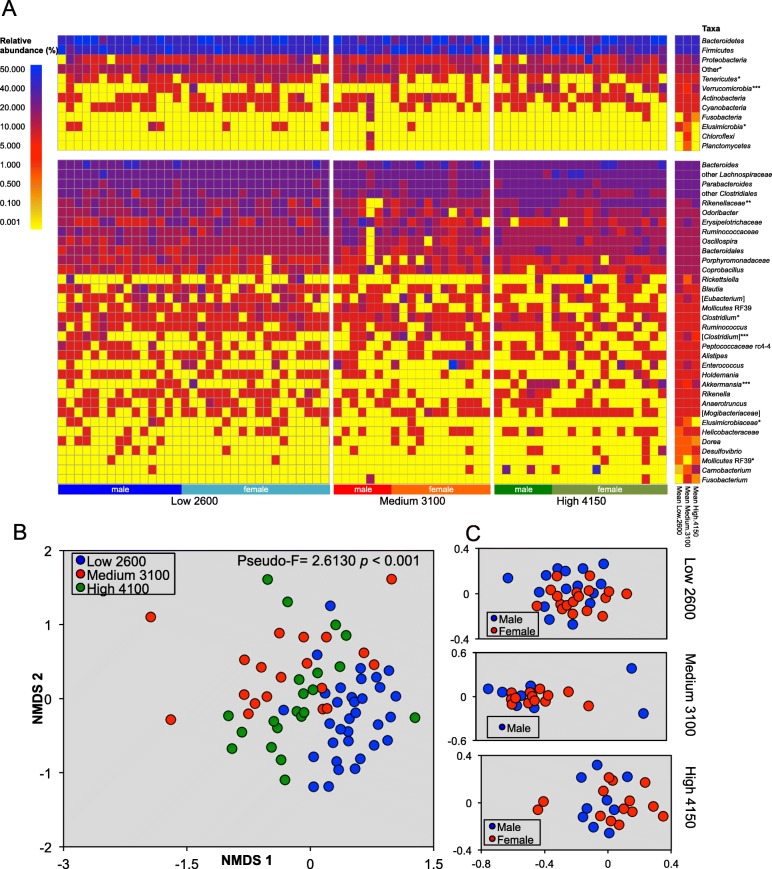
Bacterial biota in the feces of *Sceloporus grammicus* Wiegmann found at 2600 m, 3100 m, and 4150 m as determined by 16S rRNA gene barcode. Heat-map of the relative abundance of the most abundant taxonomic groups (**a**), non-metric dimensional analysis (MDS) of the weighted UniFrac distances of the three populations (**b**), and MDS of the fecal bacterial communities of males and females (**c**). Kruskal-Wallis test (*p ≤ 0.05, **p ≤ 0.01, ***p ≤ 0.001) was used to determine the effect of altitude on the relative abundance of the different bacterial groups and perMANOVA to find differences in bacterial communities of the three populations

The relative abundance of *Verrucomicrobia* was significantly higher in the High-4150 zone than in the other zones (Fig. 3a; Table 2). At lower taxonomic levels, the relative abundance of *Elusimicrobiaceae* was significantly higher in the Low-2600 population compared to the High-4150. The relative abundance of *Paenibacillus* and *Ralstonia* was significantly higher in the Medium-3100 compared to the other altitudes, and that of *Rikenellaceae*, *Akkermasia*, *Clostridium*, and *Oscillospira* in the High-4150 population compared to the other altitudes. The fecal bacterial community composition was different significantly in the three zones as determined by a perMANOVA analysis, but sex had no significant effect (Fig. 3b, c).

**Table 2:** Relative abundances and statistics for microbial groups that differed significantly (Kruskal-Wallis test) in abundance between three populations of *Sceloporus grammicus* along an altitudinal gradient

Molecular marker	Taxonomic group	Relative abundance (%)						Post-hoc Dunn’s test^a^		
		Low-2600		Medium-3100		High-4150				
		Mean	Sd	Mean	Sd	Mean	Sd	2600 vs. 3100	3100 vs. 4100	2600 vs. 4100
Bacterial 16S rRNA	*Elusimicrobia*	0.104	0.268	0.017	0.075	0	0	1.96	0.51	2.60*
	*Tenericutes*	0.640	0.614	0.508	0.634	0.226	0.266	1.03	1.35	2.59*
	*Verrucomicrobia*	0.238	1.106	0.069	0.233	1.037	1.515	0.94	− 3.43**	− 2.92**
	*Eggerthella*	0.005	0.028	0.009	0.038	0.055	0.094	− 0.24	− 2.35*	− 2.92*
	Other *Bacteroidetes*	0.560	0.459	0.336	0.642	0.647	0.768	2.60*	− 1.83	0.50
	Other *Bacteroidales*	2.033	0.959	1.507	1.095	3.562	2.556	1.57	− 3.25**	− 2.07
	Other *Rikenellaceae*	2.311	1.613	0.999	1.025	3.133	2.327	3.19**	− 3.82***	− 1.03
	*Rikenellaceae*	2.549	1.660	1.619	1.559	3.733	3.018	2.07	− 2.94**	− 1.20
	*Elusimicrobiaceae*	0.104	0.268	0.017	0.075	0	0	1.96	0.51	2.60*
	Other *Bacilli*	0	0	0	0	0.047	0.105	0	− 2.62*	− 2.97**
	Other *Bacillales*	0.174	0.330	0.009	0.038	0.008	0.036	2.61*	0.03	2.73*
	*Paenibacillus*	0	0	0.207	0.472	0	0	− 3.96***	3.60***	0
	Other *Enterococcaceae*	0.030	0.104	0.422	1.304	0.413	0.872	− 1.16	− 1.60	− 3.01**
	Other *Clostridiaceae*	0	0	0.034	0.117	0.055	0.130	− 1.32	− 0.95	− 2.45*
	*Clostridium* (*Clostridiaceae*)	0	0	0.706	2.392	0.390	0.919	− 3.19**	0.49	− 2.73*
	*Clostridium* (*Erysipelotrichaceae*)	0.337	0.851	1.275	2.987	0.094	0.152	− 2.08	2.57*	0.76
	*Achromobacter*	0	0	0.034	0.088	0.008	0.036	− 2.40*	1.53	− 0.73
	*Ralstonia*	0	0	0.034	0.088	0	0	− 2.74*	2.49*	0
	Mollicutes RF39	0.620	0.615	0.508	0.634	0.210	0.254	0.90	1.45	2.58*
	*Akkermansia*	0.233	1.107	0.069	0.233	1.037	1.515	0.78	− 3.44**	− 3.09**
18S rRNA	*Archaeplastida*	0.004	0.023	0.002	0.005	0.057	0.128	0.26	− 2.87*	− 3.79***
	*Opisthokonta*	99.787	0.6215	96.942	10.239	98.18	3.748	− 1.64	− 1.18	3.25**
	*Bryophyta*	0	0	0.002	0.005	0.049	0.129	− 0.71	− 2.00	− 3.23**
	other *Anurofeca*	0	0	0.075	0.256	0	0	− 2.57*	2.33	0
	*Chytridiales*	0.576	1.151	0.008	0.021	0.021	0.045	1.08	− 1.68	2.91**
	*Rhizophydiales*	0.011	0.058	0.002	0.005	0.007	0.011	0.35	− 1.78	2.56*
	uncultured *Chytridiomycetes*	0.034	0.174	0.029	0.062	0.457	0.610	1.36	− 2.39	− 4.41***
	Other *Pezizomycotina*	0.015	0.055	0.008	0.021	0.123	0.293	0.06	− 2.36	− 2.80*
	*Eurotiomycetes*	6.558	13.342	0.026	0.042	0.586	0.889	3.98***	− 2.45*	1.39
	*Saccharomycetes*	5.334	19.615	8.053	26.098	12.122	19.489	− 0.18	− 2.87*	− 3.70***
	*Agaricomycetes*	0	0	0.050	0.104	0.450	1.264	− 2.52*	− 1.41	− 4.50***
	*Tremellomycetes*	0.025	0.090	0.024	0.059	3.655	9.549	0.53	− 2.13	− 3.18**
	*Mortierellales*	0.001	0.003	0.005	0.008	0.047	0.093	− 1.35	− 1.84	− 3.74***
	*Umbelopsidales*	0	0	0	0	0.067	0.148	0	− 3.69***	− 4.50***
	*Leidyana*	0	0	0	0	0.015	0.038	0	− 2.51*	− 3.06**
	*Colpodida*	0	0	0.008	0.012	0.016	0.036	− 2.45*	− 0.33	− 3.11**
	other *Poterioochromonas*	0	0	0.009	0.016	0	0	− 3.71***	3.35**	0
ITS region	*Penicillium thomii*	0.020	0.064	0	0	1.425	3.495	0.51	− 2.47	− 2.57*
	*Humicola grisea*	0	0	0	0	0.048	0.096	0	− 1.94	− 2.48*
	*Goffeauzyma gilvescens*	0	0	0	0	1.159	2.758	0	− 1.94	− 2.48*
	*Naganishia friedmannii*	0	0	0	0	0.411	1.153	0	− 1.94	− 2.48*
	*Trichosporon insectorum*	0	0	0	0	0.097	0.158	0	− 2.41*	− 3.08**
	*Malassezia globosa*	0	0	0	0	0.121	0.290	0	− 1.94	− 2.48*
	*Basidiobolus ranarum*	21.413	12.012	11.957	6.552	0.290	0.307	1.66	1.99	4.36***

^a^Significant differences are in bold and asterisks denote *p* values where *p < 0.05, **p < 0.01, and ***p < 0.001

#### Micro-Eukaryotic communities

A total 843,387 good quality sequences of the 18S rRNA gene and 397 OTUs were obtained. On average, ^0^D_α_ were 41 ± 28 in the fecal samples of the Low-2600 population, 37 ± 17 in the Medium-3100, and 63 ± 31 in the High-4150 and was significantly different between Low-2600 and High-4150 population. ^1^D_α_ and ^2^D_α_, which reflects heterogeneity and evenness, were significantly higher in the High-4150 than in the Medium-3100 (Fig. 2).

**Fig. 4:**
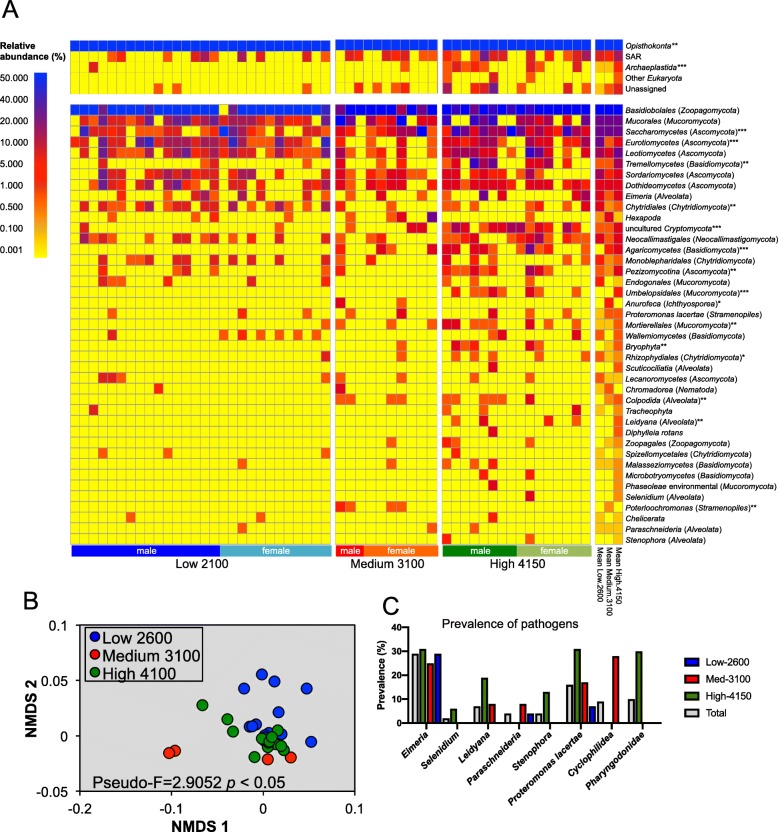
Micro-Eukaryotes in the feces of *Sceloporus grammicus* Wiegmann found at 2600 m, 3100 m, and 4150 m as determined by 18S rRNA gene barcode. Heat-map of the relative abundance of the most abundant taxonomic groups (**a**), non-metric dimensional analysis (MDS) of the weighted UniFrac distances of the three populations (**b**), and prevalence of protist pathogens in the different populations (**c**). Kruskal-Wallis test (*p ≤ 0.05, **p ≤ 0.01, ***p ≤ 0.001) was used to determine the effect of altitude on the relative abundance of the different micro-eukaryotic groups and perMANOVA to find differences in eukaryotic communities of the three populations

The foremost eukaryotic supergroup was *Opisthokonta* with relative abundance of 99.5 ± 0.5%, but members of SAR (0.4 ± 0.5%) and *Archaeplastida* (0.2 ± 0.2%) were also detected. The *Opisthokonta* supergroup includes Fungi and the multicellular kingdom of animals (Metazoa). *Archaeplastida* includes green plants and red algae while the SAR supergroup consists of *Stramenopiles* (diatoms, kelps, and oomycetes), *Alveolata* (ciliates, dinoflagellates, and parasitic apicomplexans), and *Rhizaria* (foraminifera, filose amoebae, and heterotrophic flagellates with filose pseudopodia). At lower taxonomic levels, the most abundant families were *Basidiobales* (72.2 ± 32.4%) and *Mucorales* (8.6 ± 19.1%) belonging to the *Mucoromycota* division and *Saccharomycetes* (7.8 ± 20.9%) and *Eurotiomycetes* (3.4 ± 9.8%) from the *Ascomycota* division (Fig. 4a). Although the 18S rRNA metabarcode detected mostly Fungi, protist, such as *Proteromonas lacertae* and apicomplexan parasites, such as *Stenophora*, *Selenidium*, *Leidyana*, *Paraschneideria* and *Eimeria*, and plants (*Tracheophyta*) and mosses (*Bryophyta*) were also identified. The relative abundance of *Eurotiomycetes* was significantly higher in the Low-2600 population than in the other populations (Table 2). *Poterioochromonas* was only detected in the Medium-3100 population, and uncultured *Chytridiomycetes*, *Umbelopsidales*, *Saccharomycetes*, *Bryophyta*, *Agaricomycetes*, *Tremellomycetes*, and *Leidyana* were significantly higher in the High-4150 population. Overall, the microscopic Eukaryotic community structure in the feces of the lizards was different significantly between the three altitudes (Fig. 4b).

A high relative abundance of the tapeworm *Cyclophyllidea* (24.5 ± 11.7) was found in the Medium-3100 population and a nematode member of the *Pharyngodonidae* family (0.8 ± 2.1) in the High-4150 population in the metabarcoding analysis of the gene *cox*I (Additional file 1: Table S1). No parasites were detected in the feces of lizards found at lower altitudes.

#### Fungal communities

A total of 350,472 good quality sequences of the ITS1-5.8S-ITS2 region were obtained grouping 1547 OTU-97%. ^0^D_α_ were 29 ± 8 in the fecal samples of the Low-2600 population, 17 ± 9, in the Medium-3100, and 23 ± 7 in the High-4150. True diversity at all *q* orders (^0^D_α_, ^1^D_α_, and ^2^D_α_) was significantly higher in the feces of the lizard population in the Low-2600 altitude than in the Medium-3100 (Fig. 2).

**Fig. 5:**
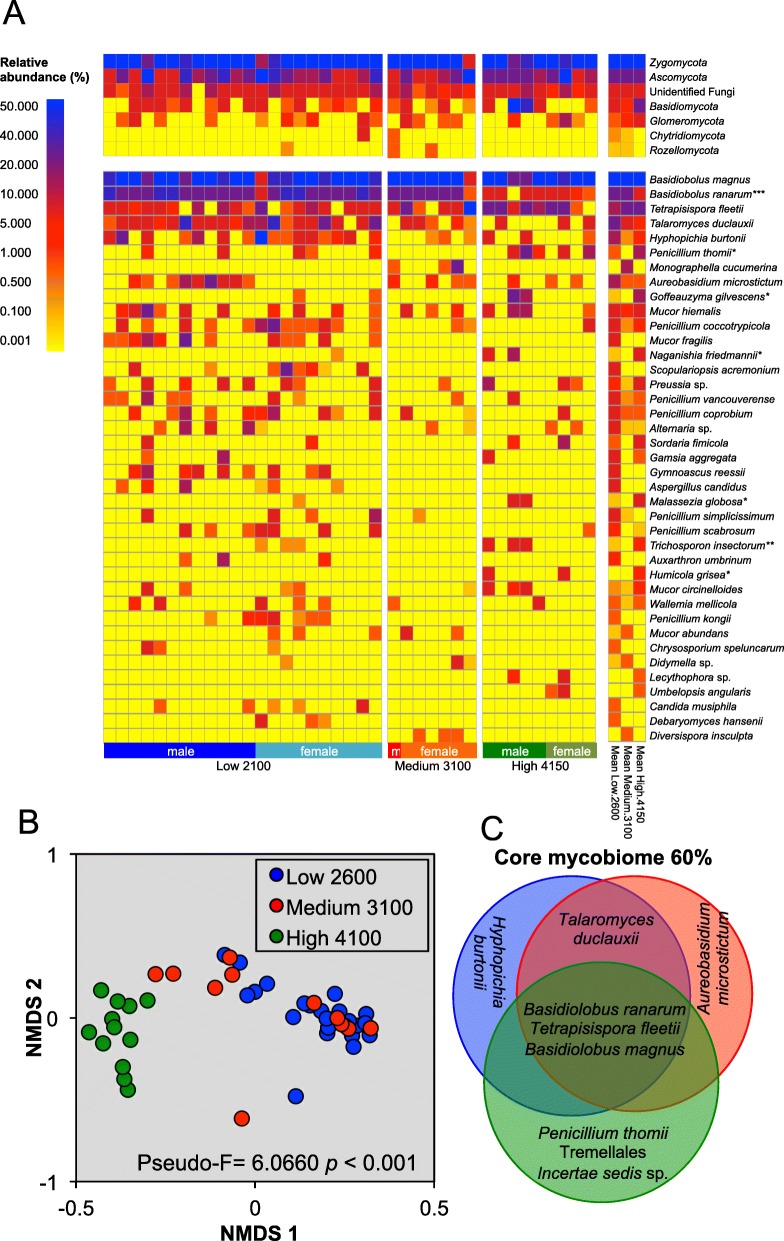
Fungi in the feces samples of *Sceloporus grammicus* Wiegmann found at 2600 m, 3100 m, and 4150 m as determined by ITS region barcode. Heat-map of the relative abundance of the most abundant taxonomic groups (**a**), non-metric dimensional analysis (MDS) of the Bray-Curtis distances of the three populations (**b**), and Venn diagram of the species of the core mycobiota (**c**). Kruskal-Wallis test (*p ≤ 0.05, **p ≤ 0.01, ***p ≤ 0.001) was used to determine the effect of altitude on the relative abundance of the different fungal groups and perMANOVA to find differences in fungal communities of the three populations

*Zygomycota* was the most abundant fungal division (relative abundance 76.7 ± 29.4%), followed by *Ascomycota* (18.6 ± 3.2%) and *Basidiomycota* (3.3 ± 14.3%) (Fig. 5a). *Glomeromycota*, *Chytridiomycota*, and *Rozellomycota* were also detected, although with a relative abundance < 1%. The most abundant species were *Basidiobolus magnus* (62.0 ± 27.0%), *Basidiobolus ranarum* (14.7 ± 13.1%), *Tetrapisispora fleetii* (4.8 ± 16.3%), and *Talaromyces duclauxii* (3.6 ± 10.3%). The relative abundance of *B*. *ranarum* was significantly higher in the Low-2600 and Medium-3100 populations than in the High-4150 population, while that of *Trichosporon insectorum*, *Penicillium thomii*, *Humicola grisea*, *Goffeauzyma gilvescens*, *Naganishia friedmannii*, and *Malassezia globosa* were significantly higher in the High-4150 than in the population at lower altitudes (Table 2).

Fungal sequences of the *cox*I gene of six genera of *Ascomycetes*, two of *Basidiomycetes*, and one (*Lichtheimia ramosa*) of *Zygomycetes* were also retrieved. Six of them, i.e., *Penicillium* spp. (*P*. *brevicompactum*, *P*. *coprobium*, *P*. *chrysogenum*, *P*. *commune*, *P*. *crustosum*, and *P*. *griseofulvum*), *Leohumicola*, and *Rhodotorula* were significantly higher in the feces of the lizards found in the Low-2600 than in the Medium-3100 and High-4150 population, while *Candida*, *L*. *ramosa*, and *P*. *citrinum* were significantly higher in the High-4150 population compared to the other two populations (Additional file 1: Table S1).

The overall structure of the fecal fungal communities, based on the ITS1 analysis, was significantly different between the Low-2600 and High-4150 *S. grammicus* populations (Fig. 5b).

### Functional profiles of bacterial communities associated with *Sceloporus grammicus*

**Fig. 6:**
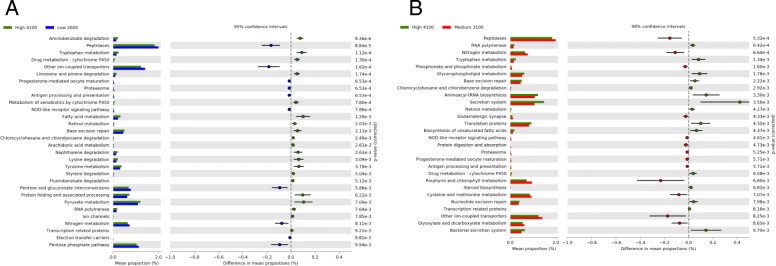
KEGG orthologs classification of the predicted functions of the bacterial communities in the fecal samples of *Sceloporus grammicus* Wiegmann found at 2600 m, 3100 m, and 4150 m. Comparison between the populations at 2600 m and 4150 m (**a**), and comparison between the populations at 3100 m and 4150 m (**b**)

The prediction of the functional profile of bacterial communities expressed in terms of functional orthologs using the KO database revealed that 47.8% of the KOs were assigned to the KEGG metabolism pathway, 18.3% to genetic information processing, 15.6% to environmental information processing, 3.1% to cellular processes, and 0.7% to organismal systems (Additional file 1: Table S3). Within KEGG metabolism pathway, KOs were grouped in the modules of carbohydrates (11.9%), amino acids (9.2%), energy (5.6%), cofactor and vitamins (3.9%), nucleotides (3.8%), and lipid metabolism (3%). Several functions were different significantly between populations of the High-4150 and Low-2600, and High-4150 and Medium-3100 populations (Fig. 6). However, no difference in functionality of the microorganisms was found in the feces of Low-2600 and Medium-3100 lizard populations. For example, peptidases and nitrogen metabolism-related KOs were significantly higher in the Low-2600 and Medium-3100 populations compared to those in High-4150. The High-4150 microbiome contained a highly significant relative abundance of KOs related with metabolism of aminoacids, vitamins and vitamins precursors, membrane components, and key intermediates of metabolic pathways, such as tryptophan, tyrosine, aminobenzoate, retinol, fatty acids, arachidonic acid, glycerophospholipids and pyruvate, and several aromatic compounds degradation and xenobiotics metabolism via cytochrome P450.

### *cox*I metabarcoding analysis of feces of *Sceloporus grammicus* along an altitudinal gradient

The 41 fecal samples (15 from the Low-2600, 13 from the Medium-3100, and 13 from the High-4150 populations) retrieved 127,795 high-quality gene *cox*I sequences. The sequences were clustered in 669 OTU-97%. Of all sequences, 40.9% belonged to *Arthropoda*, 7.0% to *Ascomycota*, 50.6% to *Zygomycota*, while 0.8% of the sequences remain unidentified (Additional file 1: Table S1). Four classes, i.e., *Arachnida* (four families), *Chilopoda* (one family), *Insecta* (24 families), and *Malacostrata* (one family), and 30 families were identified belonging to *Arthropoda* (Table 3). Of them, 25 families were found in the feces of the Low-2600, 11 in the Medium-3100, and only two (*Eremaeidae* and *Gryllidae*) in the High-4150 population.

**Table 3:** Taxonomic assignation of the *cox*I gene sequences belonging to *Arthropoda* of the DNA from fecal samples of *Sceloporus grammicus* along an altitudinal gradient

Taxon	2600 masl	3100 masl	4150 masl
*Arachnida*	1.87^a^	1.30	0.25
*Araneae*	0.04	0.00	0.00
*Araneidae*	0.04	0.00	0.00
*Sarcoptiformes*	1.83	1.30	0.25
*Acaridae*	0.06	1.11	0.00
*Eremaeidae*	0.00	0.00	0.25
*Histiostomatidae*	0.00	0.19	0.00
*Chilopoda*	1.45	0.00	0.00
*Geophilomorpha*	1.45	0.00	0.00
*Geophilidae*	1.45	0.00	0.00
*Insecta*	52.80	24.38	0.15
*Coleoptera*	9.73	13.90	0.00
*Carabidae*	0.16	0.17	0.00
*Chrysomelidae*	0.02	0.22	0.00
*Curculionidae*	3.59	2.83	0.00
*Lycidae*	0.15	0.00	0.00
*Tenebrionidae*	5.82	10.68	0.00
*Diptera*	0.34	0.89	0.00
*Calliphoridae*	0.21	0.00	0.00
*Limoniidae*	0.02	0.33	0.00
*Sciaridae*	0.11	0.22	0.00
*Hemiptera*	4.11	8.93	0.00
*Anthocoridae*	0.07	2.33	0.00
*Cercopidae*	2.63	0.00	0.00
*Cicadellidae*	0.18	0.00	0.00
*Cydnidae*	0.00	2.73	0.00
*Cymidae*	0.49	0.00	0.00
*Membracidae*	0.11	0.00	0.00
*Miridae*	0.28	0.00	0.00
*Ortheziidae*	0.27	0.00	0.00
*Hymenoptera*	0.16	0.53	0.00
*Formicidae*	0.10	0.53	0.00
*Halictidae*	0.01	0.00	0.00
*Ichneumonidae*	0.05	0.00	0.00
*Lepidoptera*	0.07	0.12	0.00
*Erebidae*	0.04	0.00	0.00
*Gelechiidae*	0.03	0.00	0.00
*Orthoptera*	36.66	0.01	0.14
*Gryllidae*	36.66	0.01	0.14
*Psocodea*	1.68	0.00	0.00
*Myopsocidae*	1.63	0.00	0.00
*Psocidae*	0.05	0.00	0.00
*Malacostraca*	0.00	0.18	0.00
*Isopoda*	0.00	0.18	0.00
*Desmosomatidae*	0.00	0.18	0.00

^a^Relative abundance of the *cox*I gene sequences assigned to the taxa

## Discussion

The intestinal microbiota is a complex network of bacterial, fungal, protistan, archaeal, and viral communities that play an important role in the well-being of its host. The intestinal microbiota of reptiles has been studied little and in less so under natural conditions. The microbiota composition, however, is highly relevant as it might be related to ecophysiological adaptations of ectotherms to environmental changes in the context of climate change and might help their conservation [6].

The core bacterial community of the gastrointestinal tract and feces of *S*. *grammicus* was dominated by *Firmicutes* and *Bacteroidetes*. In general, the vertebrates’ gastrointestinal tract harbors a similar and conservative bacterial assemblage dominated by *Firmicutes*, *Bacteroidetes*, and *Proteobacteria*, with low relative abundances of other bacterial phyla [41]. The fecal microbiota of reptiles, such as herbivorous lizards, iguanas, and tortoises [42, 43], the Burmese phyton (*Python molurus*) [44], and the lizards *Phrynocephalus vlangalii* [18], *Liolaemus parvus*, *Liolaemus ruibali*, and *Phymaturus williamsi* [45] are dominated also by *Firmicutes* and *Bacteroidetes*. However, it has been shown that the bacterial assemblages in the gut of squamates are affected by different parameters, such as diet, captivity, digestion of particular prey items, and periods of fasting [6, 45–48]. Additionally, the bacterial communities are different across gut regions [45–47]. For instance, the large intestine of the Burmese phyton was dominated by *Bacteroidetes* during fasting, while *Firmicutes* dominated during active digestion [44]. Kohl et al. [45] found that the composition of the bacterial communities in hindgut of three species of lizards was similar to that in their feces. They suggested that feces are an acceptable indicator for microbial diversity in the gut. A similar observation was found in this study. However, our conclusion was limited as we did not analyze the bacterial community across the gut regions and the sample size was small (*n* = 4).

*Bacteroides* and *Parabacteroides* were the most abundant genera in the gastrointestinal tract and feces of *S*. *grammicus* as found often in birds, mammals, reptiles, and insects [e.g., 8]. Members of *Bacteroides* participate in the degradation of biopolymers, mainly polysaccharides, and *Bacteroides thetaiotaomicron* regulates intestinal genes involved in absorption of nutrient and intestinal maturation [49].

A high relative abundance of *Zygomycota* and *Ascomycota* was detected in the feces of *S*. *grammicus* using different DNA barcodes, i.e., ITS region, 18S rRNA, and *cox*I. Fungi have been determined little in the gut or feces of vertebrates, even in humans. However, they have a great relevance in the symbiotic relation with the host as they have the capacity to degrade complex molecules, and participate in the fermentation and production of secondary metabolites. The core fecal mycobiome of *S*. *grammicus* was composed of *Basidiobolus ranarum*, *B*. *magnus*, and *Tetrapisispora fletii. Basodiobolus* belongs to *Entomophthoromycota* (*Zygomycota*) and is a parasite-pathogen of arthropods and insects that use subtilisin-like serine proteases to degrade chitin-associated proteins in the insect procuticle [50]. It is possible that lizards ingest propagules of *Basidiobolus* spp. through infected insects or carrying their conidia [51]. The association of *Basidiobolus* spp. with the vertebrate gut, particularly reptiles and amphibians, has been reported before [52, 53]. Strains of *Basidiobolus* with extracellular chitinase production have been isolated from the frogs’ intestine [54]. It is possible that reptiles and amphibians obtained *Basidiobolus* spp. from their diet. A commensal relationship of *Basidiobolus* spp. with *S*. *grammicus* might be linked to the chitinolitic capabilities of the fungi participating in the degradation of the exoskeleton of the arthropods that the lizard feeds on. In humans, a strong link exits between the food consumed and fungal abundance in the gut [55]. Fermentative yeasts are also an integral part of the gut mycobiota. The yeast *Tetrapisispora* has been associated with insects, particularly cockroaches [56, 57]. *Tetrapisispora phaffii* produces a killer toxin (glycoprotein Kpkt) that is lethal to other spoilage yeasts [58].

Gut Protozoa and Helminthes have been considered parasites and pathogens. However, the eukaryotic residents of the gut are often commensals and many gut Protozoa play an important role in controlling bacterial populations [59]. No core group of eukaryotic residents was detected, however, in the feces of *S*. *grammicus*.

### Diet of *Sceloporus grammicus* along an altitudinal gradient

*Sceloporus grammicus* has been described as an insectivore species with a tendency to feed on *Coleoptera* and *Hymenoptera* [12]. In this study we found that *S*. *grammicus* feeds mainly on *Orthoptera* and *Coleoptera* insects. Here, we confirm that with altitude the number of arthropods that *S*. *grammicus* ingests and their diversity sharply decreased. The decrease was dramatic; of the 25 families of arthropods found in the feces of the Low-2600 population, only two families were detected in the High-4150 population, and the relative abundance of *cox*I belonging to arthropods dropped from 55.7% to only 0.4%. It is well known that species richness of most arthropods and their body size decreases with increasing elevation (e.g., [60, 61]). Additionally, low temperatures limited the thermal opportunities for foraging activity of lizards at high elevations. This implies that lizards at high elevations must (1) be better at extracting the available energy from their diet, (2) reduce expenditure from their total energy budget, or (3) allocate less energy to growth. In the first scenario, the gastrointestinal microbial community must play a crucial role, while for the third scenario, Sears [62] found that a high elevation population of *Sceloporus graciosus* grew faster than populations at lower altitude at the expense of their metabolic expenditure.

### Composition of the fecal microbiota of *Sceloporus grammicus* along an altitudinal gradient

*Sceloporus grammicus* is exposed to extreme conditions at high altitude, e.g., 4150 m a.s.l, such as a low partial oxygen pressure, low temperatures, high level of ultraviolet radiation, and dietary restrictions. In this study, the proportion of *Akkermansia* (*Verrucomicrobia*) increased in the feces of *S*. *grammicus* at high altitude. *Akkermansia* spp*.* are mucin-degrading bacteria that live in the mucus layer of the intestine [63]. In other animal models, the proportion of *Akkermansia* decreases with altitude, for example in the lizard *P*. *vlangalii* [18] and in the wild house mice [64]. The Tibetan antelope (*Pantholops hodgsonii*), which is very well adapted to high altitudes, with a vegetarian diet low in calories and vitamins also contains large proportions of *Akkermansia* [65]. Several studies found that *Akkermansia muciniphila* is highly competitive in hosts with restricted diets low in calories and nutrients as it is capable of subsisting on host mucus as a sole source of carbon and nitrogen [66]. For example, its abundance increased after fast in hamsters [67] and the Burmese phyton [44].

Similarly, the proportion of *Oscillospira* increased in the High-4150 population. *Oscillospira* has never been cultivated, so little is known of its ecological role or physiological properties in the intestinal tract; yet is frequently detected in metagenomic studies of vertebrate intestinal biota [68]. Kohl et al. [69] compared the response of gut microbiota of different vertebrates to fasting and the relative abundance of *Oscillospira* increased in the cecum of a bird, a fish, and a mammal during fasting. They speculated that *Oscillospira* degrade glycans of the host, such as fucose, sialic acids, and glucuronic acid. As such, it is more likely that dietary restriction enrich members of *Oscillospira* living at 4150 m instead of low O_2_ pressure and low temperature as suggested by Zhang et al. [18]. Additionally, the lizards at this high altitude were exposed to longer periods of lower temperatures than those at lower altitude, which promotes long periods of inactivity and fasting [15].

The relative abundance of *Oscillospira* and *Clostridium*, and members of *Rikenellaceae* and *Ruminococcaceae* increased in the feces of lizards from the High-4150 and the Medium-3100 populations. These bacterial groups are reported as specialists in the digestion of cellulose and are playing an essential role in the fermentation of fiber in herbivorous, including reptiles [42, 45, 70–72]. A significant number of reads of 16S rRNA of chloroplast in lizards from the High-4150 populations and in a minor proportion in the Medium-3100 were detected. These data are not shown as NGS quality good practices suggest eliminating reads from chloroplast and mitochondria (https://galaxyproject.github.io/training-material/topics/metagenomics/tutorials/mothur-miseq-sop/tutorial.html). Metabarcoding using 18S rRNA revealed also a significantly higher proportion of *Bryophyta* in the High-4150 *S*. *grammicus*. Serrano-Cardozo et al. [73] found plant material in the gastrointestinal tract of *Sceloporus* spp. in a semiarid region of Mexico. It is possible that (1) plant tissue was accidentally digested during the capture of prey, (2) plant material originates from the intestinal content of the prey, (3) ingestion of plants might be an additional source of water, and/or (4) plant material was ingested deliberately by members of the High-4150 population as the amount of insects that can serve as food is limited. The gut biota of High-4150 *S*. *grammicus* was enriched with microorganisms specialized in plant fiber degradation (*Clostridium*, *Rikenellaceae*, and *Ruminococcaceae*), which would suggest that plant material was ingested deliberately. The gut bacteriome predicted functions showed also a significantly higher abundance of functions related to the degradation of several aromatic compounds and xenobiotics in the High-4150 population. This suggests that plant material was ingested deliberately and the intestinal associated bacterial biota have the capacity to degrade vegetal material and detoxify the aromatic compounds of the vegetal material.

Fungi are known to produce a diverse array of secondary metabolites. However, little is known about their contribution to the gut ecology. The relative abundance of *B*. *ranarum* was significantly higher in the Low-2600 and Medium-3100 populations than in the High-4150, but that of *B*. *magnus* was similar. *Basidiobolus magnus* prefers nutrient poorer substrates than *B*. *rararum* [74] while *B*. *ranarum* is also a pathogen in humans as it can grow at 37 °C [51]. It is likely that the low temperature at high altitude was unfavorable for *B*. *ranarum* and that *B*. *magnus* was more competitive in the gut of the High-4150 population.

The feces of the High-4150 population contained biome microorganisms of insects, e.g., *Trichosporon insectorum* and several *Gregarinasina* (*Leidyana*, *Selenidium*, and *Stenophora*) but a low number of *cox*I from insects. *Trichosporon insectorum* is a basidiomycete yeast and the resident of the gut of insects [75], while *Gregarinasina* are intracellular parasitic apicomplexan alveolates found in the intestinal epithelial cells of cockroaches, mealworms, grasshoppers, crickets, crayfishes, and centipedes [76]. We hypothesize that the High-4150 population extracts as much nutrients from their food as possible by maintaining it longer in their lumen. Many studies on vertebrates have shown that the production of digestive enzymes increased with substrate availability in the gut lumen [77]. However, increasing enzymatic and absorptive capacities is limited and correlated to the amount of food digested because of its costs.

Little is known about the role of Fungi in the gut ecosystem of reptiles. Gouba and Drancourt [78] found 221 different fungal species belonging to the phyla *Ascomycota*, *Basidiomycota*, and *Zygomycota*, including *Basidiobolus ranarum*, *Penicillium* spp., and *Aspergillus* spp. in the intestinal human microbiota. Hallen-Adams and Suhr [79] reported that only a limited number of fungal species, mostly *Candida* yeasts, are capable to colonize and grow in the gut of humans. In this study, different assemblages of fungal species were found in the feces of the three populations of *S*. *grammicus*. In the Low-2600 population, the assemblage contained members of *Aspergillus*, *Eurotiomycetes*, *Talaromyces*, and several *Penicillium* spp. and in the High-4150 population members of *Penicillium* spp., *Candida*, *Goffeauzyma*, *Naganishia*, and *Malassezia* yeasts*.* In the Med-3100 population, the fungi were mainly members of *Candida*. It is possible that *Penicillium* species contributed to the gut ecosystem as they are producers of secondary metabolites, extracellular enzymes (alginase, endoglucanase, β-glucosidase), and bioactive compounds (anti-tumor, anti-fungal, and antibacterial activity), while some species possess fatty acid synthases that fulfil numerous central biological roles in living cells [80, 81]. It is possible that the different assemblages of yeast and anamorphic fungi might be the result of the environments that the different populations inhabit. For example, *Goffeauzyma* and *Naganishia* yeast have been reported as psychrophilic and found in extreme cold environments [82, 83].

In the High-4150 populations, a higher relative abundance of *Agaricomycetes* was found compared to the other two populations. We discard the possibility of spore contamination of the fecal samples during the collection as all samples were collected with the same cautious and under sterile conditions. It is possible that *Agaricomycetes* derived from the intestines of the insects. However, although fungivorous reptiles have never been reported, it is still possible that the limiting food resources of the High-4150 population might have obliged the lizards to exploit different food resources. Kohl et al. [45] investigated environmental sources that might contribute to the gut microbial communities of wild omnivorous lizards. They found that soil bacteria and the invertebrate diet did not contribute significantly to the gut communities of lizards, but the type of plants consumed did. The same was found in herbivorous desert wood rats (*Neotoma lepida*) [84]. There was substantial overlap between the gut microbiota of desert wood rats and the phyllosphere microbiota of their dietary plants. In the Burmese pythons, the microbes of the rodents they consumed contributed < 1% to their gut community [44].

### Predicted fecal bacteriome functions of *Sceloporus grammicus* along an altitudinal gradient

We are aware that the PICRUSt analysis in non-model organisms should be interpreted with care. However, we found that the abundance of functional features associated with metabolism were different between the lizard populations. Peptidases and nitrogen metabolism were higher in the Low-2600 and Medium-3100 lizard populations compared to High-4150 ones, while metabolism of aminoacids, vitamins, and key intermediates of metabolic pathways were higher in the High-4150 bacteriome. We hypothesize that the difference in quality and quantity of the diet of *S*. *grammicus* at different altitudes affected the functional profiles of their gut bacteriome. Similarly, Wang et al. [85] found that artificially fed Bar-headed geese had a higher bacterial gene content related to carbohydrate transport and metabolism, energy metabolism and coenzyme transport, and metabolism, compared to the wild ones.

### Alpha and beta diversity of the fecal microbiota of *Sceloporus grammicus* along an altitudinal gradient

Although the microbial composition varied greatly among individuals, the community composition of bacteria, micro-eukaryotes, and fungi in the feces of three populations of *S*. *grammicus* was different along the altitudinal gradient. In an experimental study with the lizard *Z*. *vivipara*, species richness of the gut bacterial biota decreased when they were maintained at a 2 to 3 °C higher temperature [6]. In *S*. *grammicus*, the bacterial diversity (^1^D_α_ and ^2^D_α_) and richness (^0^D_α_) were similar along an altitudinal gradient. The Low-2600 population had the highest ITS phylotypes diversity compared to the other populations. We can assume that the main functions of fungal communities in the gut of the Low-2600 are digestive and hydrolytic and that a high food intake promoted their high diversity. However, diversity and richness of ITS and 18S rRNA phylotypes were in general lower in the Medium-3100 population. The individuals of the Medium-3100 population had the lowest survival rates [15]. It is likely that the limited diversity of the microbiota was related with the low survival of the Medium-3100 population.

### Eukaryotic gut biota: friends or foes?

Research on parasitic infections in wildlife has received increased attention for their role in extirpations and extinctions, e.g., in amphibian. Protozoa and fungi can cause severe illness. Yet many infections are often asymptomatic, probably reflecting a long co-evolutionary history. The thin line between gut pathogen and gut commensal is hard to determine as host-parasite interactions are complex, and intestinal microbiota is possibly the source of several infections [78]. Immune responses in ectothermic vertebrates are linked to ambient temperature, but the physiological activities of pathogens also [86]. It is therefore difficult to associate the presence/prevalence of commensals and/or parasites, e.g., *Proteromonas lacertae*, *Eimeria*, *Lichtheimia*, *Pharyngodonidae* nematode, and tapeworm *Cyclophyllidea*, in the different populations of *S*. *grammicus* with health or disease. *Proteromonas lacertae* (14% prevalence in *S*. *grammicus*) is a strict anaerobic stramenopile that lives as a commensal in the posterior intestinal tract of lizards [87]. Its closest relative and human/mammal counterpart is *Blastocystis*, which can be found with high prevalence in healthy populations [88]. Members of *Lichtheimia* spp. are ubiquitously distributed fungi and saprobic decomposers of decaying organic matter in soil. Recently, they have been found to be an important emerging human pathogen and they are the second most common cause of mucormycosis in Europe and the third worldwide [51]. *Lichtheimia* was found with high frequency in the High-4150 population. *Eimeria* (7% of prevalence in *S*. *grammicus*) is a genus of apicomplexan parasites that includes various species capable of causing coccidiosis in vertebrates. *Pharyngodonidae* nematodes (10% of prevalence in *S*. *grammicus*) have been found parasitizing the large intestine of amphibian and reptiles [89, 90]. The cestode *Cyclophyllidea* (9% of prevalence in *S*. *grammicus*) uses a variety of insects as intermediate host. Ingestion of its eggs results in the development of a cysticercoid in the hemocoel that is infective to the ultimate host [91]. Particularly, the prevalence of this parasite is high in the Medium-3100 population, which might be also related with their low survival rates. Recent studies suggest that the environment alters the susceptibility to infections so that host infections depend on environmental conditions [92]. In general, the High-4150 population had the highest proportions of pathogens.

## Conclusions

The bacterial phyla *Firmicutes* and *Bacteroidetes* and the genera *Bacteroides* and *Parabacteroides* dominated the core fecal bacteriome of *S*. *grammicus*. The fungal phyla *Zygomycota* and *Ascomycota* and the species *Basidiobolus ranarum* and *B*. *magnus* dominated the core fecal mycobiome. The diversity and quantity of the diet decreased dramatically for the lizards at high elevations. Considering the differences in diet, it was not surprising that the composition of the main microbial groups in the feces of *S*. *grammicus* was different at the three elevations, but not between female and male lizards. It is possible that dietary restriction in *S*. *grammicus* living at 4150 m explained the high fecal abundance of *Akkermansia* and *Oscillopira*, and the low temperature enriched *B*. *magnus* in the gut of the High-4150 population. We detected important differences in the potential functions of the fecal bacteriome of *S*. *grammicus* in the three populations.

The bacterial diversity and richness were similar in *S*. *grammicus* along the altitudinal gradient. However, the Low-2600 population had a higher ITS phylotypes diversity than the two other populations and the main functions of its fungal community were digestive and hydrolytic as sufficient food intake promoted fungal diversity. We assume that the low survival rates of the Medium-3100 population might be related to the high prevalence of *Cyclophyllidea* and the low diversity of their resident microbiota.

## Supplementary information

**Additional file 1: Table S1.** Relative abundances and statistics for taxonomic groups identified through metabarcoding of the gene *cox*I that differed significantly (Kruskall-Wallis test) in abundance between three populations of *Sceloporus grammicus* along an altitudinal gradient.

## Data Availability

All sequencing data are available from the National Center for Biotechnology Information (NCBI) Sequence Read Archive (SRA) under accession number PRJNA544140.
