# Supplementary material for: Dietary effects on gut microbiota of the mesquite lizard Sceloporus grammicus (Wiegmann, 1828) across different altitudes
Source: Microbiome. 2020 Jan 24;8:6. doi: 10.1186/s40168-020-0783-6 (PMC6982387; doi:10.1186/s40168-020-0783-6)
Supplement: Supplementary file 1 — Additional file 1: Table S1. Relative abundances and statistics for taxonomic groups identified through metabarcoding of the gene coxI that differed significantly (Kruskall-Wallis test) in abundance between three populations of Sceloporus grammicus along an altitudinal gradient. [file 40168_2020_783_MOESM1_ESM.docx]

Table S1. Relative abundances and statistics for taxonomic groups identified through metabarcoding of the gene *cox*I that differed significantly (Kruskall-Wallis test) in abundance between three populations of *Sceloporus grammicus* along an altitudinal gradient.

| Taxonomic group^a^ | Relative abundance (%) | | | | | | Post-hoc Dunn’s test^a^ | | |
| --- | --- | --- | --- | --- | --- | --- | --- | --- | --- |
|  | Low-2600 | | Medium-3100 | | High-4150 | | 2600 vs 3100 | 3100 vs 4100 | 2600 vs 4100 |
|  | Mean | Sd | Mean | Sd | Mean | Sd |  |  |  |
| *Arthropoda* | 56.640 | 40.160 | 34.208 | 37.008 | 1.038 | 2.650 | 1.08 | **-3.17**** | **-4.24***** |
| *Nematoda* | 0.000 | 0.000 | 0.000 | 0.000 | 0.878 | 2.180 | 0 | **2.65*** | **2.65*** |
| *Platyhelminthes* | 0.000 | 0.000 | 11.708 | 24.515 | 0.000 | 0.000 | **-2.51*** | **-2.46*** | 0 |
| *Zygomycota* | 34.533 | 33.041 | 38.828 | 38.424 | 72.356 | 32.125 | -0.07 | **2.45*** | **2.55*** |
| *Sarcoptiformes* | 1.990 | 4.752 | 0.000 | 0.000 | 0.000 | 0.000 | **3.73***** | 0 | **-3.66***** |
| *Tyrophagus putrescentiae* | 0.000 | 0.000 | 1.443 | 3.013 | 0.000 | 0.000 | **-2.84*** | **-2.79*** | 0 |
| *Histiostomatidae* | 0.000 | 0.000 | 0.611 | 1.428 | 0.000 | 0.000 | **-2.51*** | **-2.46*** | 0 |
| *Geophilidae* | 1.212 | 4.184 | 0.000 | 0.000 | 0.000 | 0.000 | **3.15*** | 0 | **-3.09*** |
| *Curculionidae* | 7.571 | 18.973 | 0.693 | 1.788 | 0.000 | 0.000 | **2.70*** | -1.30 | **-3.95***** |
| *Bothrotes plumbeus* | 7.806 | 20.948 | 0.000 | 0.000 | 0.000 | 0.000 | **4.01***** | 0 | **-3.94***** |
| *Eleodes* sp. | 2.626 | 9.708 | 0.108 | 0.405 | 0.000 | 0.000 | 2.02 | -0.55 | **-2.54*** |
| *Tenebrio obscurus* | 0.000 | 0.000 | 6.784 | 23.831 | 0.000 | 0.000 | **-2.84*** | **-2.79*** | 0 |
| *Hemiptera* | 0.103 | 0.199 | 6.985 | 17.383 | 0.000 | 0.000 | -1.11 | **-3.04**** | -1.94 |
| *Cercopidae* | 3.222 | 6.329 | 0.000 | 0.000 | 0.000 | 0.000 | **4.82***** | 0 | **-4.73***** |
| *Scaphytopius* sp. | 0.479 | 1.596 | 0.000 | 0.000 | 0.000 | 0.000 | **2.51*** | 0 | **-2.46*** |
| *Miridae* | 0.756 | 2.678 | 0.000 | 0.000 | 0.000 | 0.000 | **-2.84*** | 0 | **-2.79*** |
| *Formicidae* | 0.166 | 0.425 | 0.000 | 0.000 | 0.000 | 0.000 | **3.45**** | 0 | **-3.38**** |
| *Acheta domesticus* | 22.487 | 36.789 | 0.054 | 0.202 | 0.786 | 2.550 | **3.84***** | 0.50 | **-3.27**** |
| *Myopsocidae* | 4.540 | 16.913 | 0.000 | 0.000 | 0.004 | 0.013 | **2.61*** | 0.56 | -1.99 |
| *Pseudomesus brevicornis* | 0.010 | 0.025 | 0.480 | 0.672 | 0.000 | 0.000 | **-2.55*** | **-3.49**** | -0.98 |
| *Penicillium* sp. | 0.269 | 0.575 | 0.000 | 0.000 | 0.052 | 0.178 | **2.49*** | 1.01 | -1.42 |
| *Penicillium chrysogenum* | 0.908 | 2.712 | 0.000 | 0.000 | 0.053 | 0.192 | **3.80***** | 0.50 | **-3.22**** |
| *Penicillium citrinum* | 0.002 | 0.006 | 0.000 | 0.000 | 0.062 | 0.116 | 0.51 | **2.46*** | 1.95 |
| *Penicillium commune* | 0.024 | 0.042 | 0.000 | 0.000 | 0.000 | 0.000 | **3.15**** | 0 | **-3.09**** |
| *Penicillium coprobium* | 5.194 | 8.995 | 0.040 | 0.148 | 0.040 | 0.123 | **4.76***** | 0.50 | **-4.16***** |
| *Penicillium crustosum* | 0.219 | 0.385 | 0.000 | 0.000 | 0.135 | 0.331 | **3.76***** | 1.40 | **-2.29*** |
| *Penicillium griseofulvum* | 0.036 | 0.068 | 0.000 | 0.000 | 0.000 | 0.002 | **3.03**** | 0.44 | **-2.53*** |
| *Leotiomycetes* family incertae sedis | 0.134 | 0.433 | 0.401 | 1.071 | 0.000 | 0.000 | 1.03 | -1.46 | **-2.47*** |
| *Leohumicola* sp. | 0.044 | 0.070 | 0.005 | 0.018 | 0.000 | 0.000 | **2.51*** | -0.46 | **-2.92*** |
| *Candida* sp*.* | 0.406 | 0.924 | 7.880 | 24.200 | 16.584 | 28.468 | 0.14 | **2.47*** | **2.33*** |
| *Pythium* sp. | 0.028 | 0.060 | 0.000 | 0.000 | 0.131 | 0.471 | **2.56*** | 0.62 | -1.89 |
| *Pharyngodonidae* | 0.000 | 0.000 | 0.000 | 0.000 | 0.878 | 2.180 | 0 | **2.65*** | **2.65*** |
| *Cyclophyllidea* | 0.000 | 0.000 | 11.708 | 24.515 | 0.000 | 0.000 | **-2.51*** | **-2.46*** | 0 |
| *Lichtheimia* sp. | 1.022 | 1.453 | 0.289 | 0.816 | 11.678 | 24.288 | **2.79*** | **3.92***** | 1.18 |
| ^a^ Significant differences are in bold and asterisks denote *p* values where *= *p* < 0.05, **= *p* < 0.01 and *** *p* < 0.001. | | | | | | | | | |
